# The impact of ex-post legislative evaluations: a scoping review

**DOI:** 10.1080/13572334.2022.2160289

**Published:** 2023-02-02

**Authors:** Linda J. Knap, Rob van Gameren, Valérie D.V. Sankatsing, Johan Legemaate, Roland D. Friele, Nivel

**Affiliations:** a Netherlands Institute for Health Services Research, Utrecht, the Netherlands; b Tranzo Scientific Center for Care and Wellbeing, Tilburg University, Tilburg, the Netherlands; c University of Amsterdam, Law Centre for Health & Life, Amsterdam, the Netherlands

**Keywords:** Ex-post legislative evaluation, evaluation of legislation, scoping review, impact, post legislative scrutiny

## Abstract

In various countries, laws are increasingly being evaluated by examining the effects in practice once a law enters into force. No systematic overview currently exists on the impact of these ex-post legislative evaluations. Therefore, this scoping review systematically examines the various types of impact of ex-post legislative evaluations. The studies we looked at demonstrate different types of impact that can be divided into the following seven categories: knowledge and understanding; confirmation of wellfunctioning legislation; legislative revision; influence on the legislative process; influence on the policy process; influence in the political sphere; and influence on society. The various types of impact are sometimes interrelated and can exist in various degrees. At the national and European levels, legislative revision and the tactical use of evaluation results in the political sphere, are the two most often mentioned categories. In contrast, the impact on society category is rarely mentioned.

## Introduction

Ex-post legislative evaluations, also referred to as post-legislative scrutiny,^1^ assess the functioning of legislation by examining whether the legislation works, how it works, and the effects that occur in practice after a law enters into force. These evaluation studies are conducted either systematically – e.g. on the basis of an evaluation clause in the law, a budgetary threshold or out of a general principle – or, on an *ad hoc* basis. In some literature, legislative evaluations are seen as a form of policy evaluation, leading to both being considered related (Klein-Haarhuis & Parapuf, 2016; Wollmann, 2016). However, despite the many similarities between policy evaluations and legislative evaluations, there are essential characteristics of legislative evaluations that warrant specific attention to this type of evaluation (van Aeken, 2011). This is especially true because legislation can be seen as one of the most powerful policy instruments, influenced by policy agendas, legal debates, and societal developments. Therefore, ex-post legislative evaluations can impact the policy domain, the surrounding legal debate, and society itself.

In recent decades, the evaluation of legislation has received more attention, both at the national and European Union (EU) levels, as the increasing number of laws has created a greater need to understand the effectiveness of legislation. The Organisation for Economic Cooperation and Development (OECD) also highlighted the importance of keeping the accumulated stock of legislation consistent and up-to-date (OECD, 2020). This has led to an institutionalisation of ex-post legislative evaluations (Anglmayer & Scherrer, 2020). Furthermore, the importance of contributing to better regulation through legislative evaluations is particularly recognised by legislators and policy makers (Bussmann, 2010; European Court of Auditors, 2018; The Law Commission, 2006; Winter, 2002). Expost legislative evaluations have gained prominence and are now considered an integral part of regulatory governance. Many countries, as well as the EU, have institutionalised ex-post legislative evaluations. For example, in 2013, as part of the EU better regulation concept, the European Commission (the Commission) introduced the ‘evaluate first’ principle, which means that the Commission aims to conduct an ex-post legislative evaluation before revising existing legislation (European Court of Auditors, 2018). Furthermore, the need for ex-post legislative evaluations became evident in the context of legislation enacted in response to the COVID-19 pandemic, when the urgency of the situation did not allow for an ex-ante evaluation or impact assessment and parliaments were often bypassed. Ex-post legislative evaluations can be used to examine whether emergency measures are still in the public interest and should be abolished or continued (OECD, 2021).

While there is a clear call for ex-post legislative evaluations, research on the impact of these evaluations is scattered. While an extensive and diverse body of scientific literature describes all types of actual and potential impact on parties involved at various times in the legislative process and with various degrees, this very diversity confirms the need for a comprehensive overview of its different types of impact. This scoping review therefore aims to systematically examine what types of potential and actual impact can be broadly identified, without limitation to jurisdictions, and what is known in the literature about the different types of potential and actual impact of ex-post legislative evaluations. The contributions of this study are manifold: we broaden our understanding of ex-post legislative evaluations (without limiting ourselves to national borders); we add to existing literature on legislative evaluation; and we develop the qualitative knowledge base for understanding the different types of impact.

## Research method

This study aims to present a broad spectrum of insight derived from literature on the types of impact of ex-post legislative evaluations in order to identify the available evidence on the same, and to analyse knowledge gaps in this field (Colquhoun et al., 2014). Therefore, this scoping review followed the methodological PRISMA-ScR framework (Arksey & O’Malley, 2005; Levac et al., 2010). Scoping reviews are an ideal tool to determine the scope or coverage of a body of literature on any given topic that is not yet well charted (in this case, the impact of ex-post legislative evaluations) providing an overview (broad or detailed) of the literature’s focus.

### Phase 1

In an effort to capture all relevant literature, the study started with a broad research question: *What can be found in the scientific literature about the methodology and impact of ex-post legislative evaluations*? To ensure a broad search strategy, the research question did not include any specific jurisdiction or field of law. After conducting a detailed search, the research question was narrowed (see phase 4).

### Phase 2

A search was made of the Web of Science, Worldcat and Legal Intelligence scientific databases using different search strings for an initial scope of the scientific literature (first quarter of 2021). Given that literature on this topic was expected to be scarce, no timespan was selected for the search. Identical search strings were applied per database with both English and Dutch search terms, provided with Boolean operators (AND, OR), wildcard symbol, quotation marks, parenthesis and truncation in order to improve the search strategy. We initially started with a broad search strategy followed by two more specific search strategies, one related to methodology and the other to impact. Synonyms were applied for this purpose. The final search strings are included in Table 1.

### Phase 3

**Figure 1:**
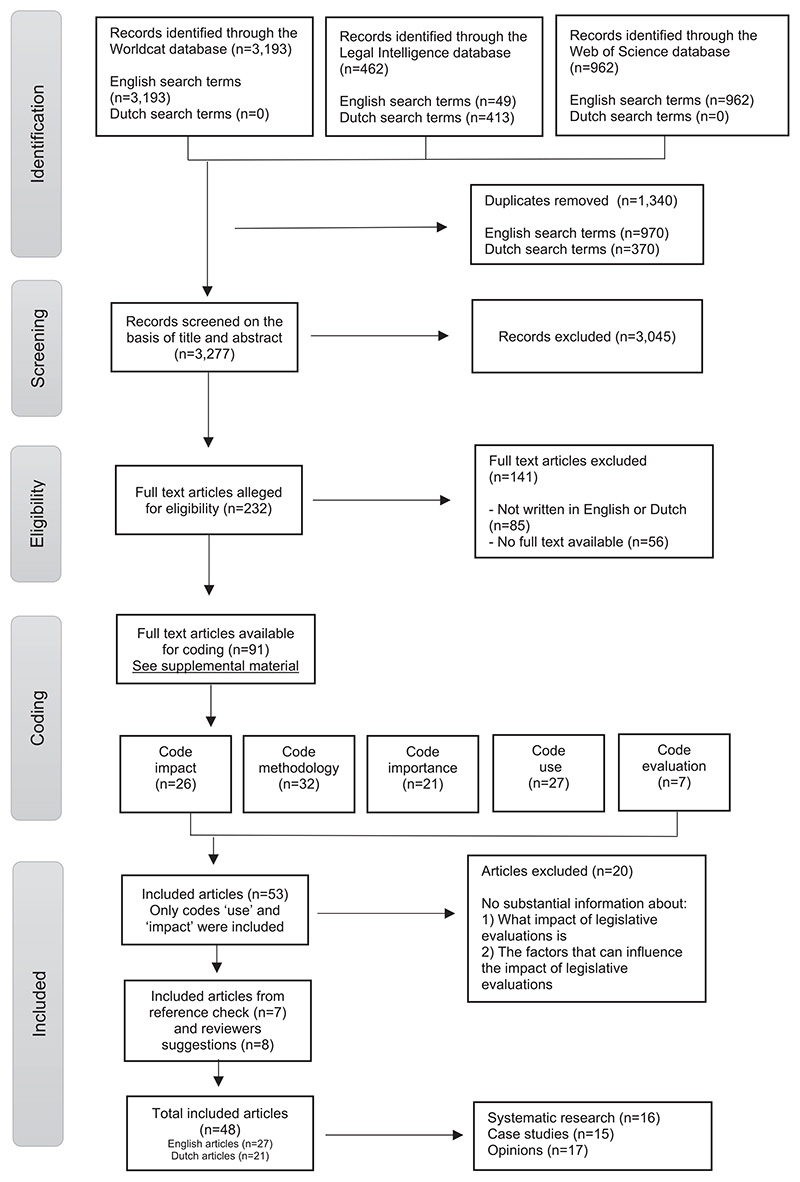
Methods flowchart.

In total, 4,204 studies were found with English search terms, and 413 studies were found with Dutch search terms (see Figure 1 for the entire search process, which is explained in more detail below). All literature was uploaded in Rayyan software, an administrative tool that facilitates the process of identifying and selecting studies when conducting a systematic literature review (Ouzzani et al., 2016). After merging the data, duplicates were removed (1,340 out of 4,617 studies). The literature (n = 3,277) was screened and selected by the first author (LK) based on title (for example, excluding titles that were not on the subject of ex-post legislative evaluations) and abstract. Independently, another author (RvG) reviewed a random selection of 5% (164 studies) using the initial criteria. For 7 of the 164 studies, disagreements between the authors about the selection had to be resolved through discussion.

Studies were deemed relevant if they focused in full or in part on the methodology and/or impact of ex-post legislative evaluations and were written in English or Dutch. Since the Netherlands has a long history of ex-post legislative evaluations and much literature is written in Dutch, the research group considered this a valuable addition to the English-language literature. During the selection of studies, those on the impact of legislation itself rather than the impact of ex-post legislative evaluations were excluded, as were studies on the impact of evaluations in general without a reference to ex-post legislative evaluations. Policy evaluations without a legal aspect were also excluded. Finally, ex-ante legislative evaluations were excluded from this study due to the different research designs used and their significance, as they are conducted before legislation is passed. On this basis, a total of 3,045 out of 3,277 studies were excluded and 232 studies were included (see Figure 1).

After selecting the studies and, in order not to miss any relevant coding, authors (LK) and (RvG) coded the studies based on title and abstract without agreeing on the codes beforehand. After, all authors agreed on the coding for the full text assessment; namely, methodology, use, impact, importance, and evaluation. Disagreements in the assigned types of coding after full text assessment were resolved through discussion between the authors. The full text versions of all 232 studies were manually searched. The studies that were either unavailable in full text (n = 56) or not written in English or Dutch (n = 85) were excluded (141 out of 232 studies). With regard to data openness, a list of the remaining 91 articles is included in the supplemental material.

### Phase 4

In phase 4, the research question was narrowed down: *What can be found in the scientific literature about the impact of ex-post legislative evaluations?* In this scoping review, impact is seen as the influence of ex-post legislative evaluations on various directly and indirectly affected parties.

As it appeared that much had already been written on the methodology of ex-post legislative evaluations, the focus on the impact of ex-post legislative evaluations could make a greater scientific contribution. For this purpose, a filtered selection was made of only studies coded with ‘impact’ (n = 26) and ‘use’ (n = 27). The 53 studies were fully read and assessed for relevance by both authors (LK) and (RvG), after which 20 studies were excluded (see Figure 1). The relevant studies (n = 33) were divided into three categories, including: systematic research in which a certain number of legislative evaluations were studied in a systematic manner (12 out of 33); case studies (12 out of 33); and expert opinions (9 out of 33). Importance was allocated in this order to the included literature and opinions could be empirically verified by case studies or systematic research. In the results of this scoping review, specific reference is elaborately made to these three research types.

References from the relevant studies were hand-searched by authors (LK) and (RvG) resulting in seven additional studies (four systematic research studies, and three expert opinion publication) that were added to the number of included studies. A final addition was made based on suggestions from reviewers of the manuscript. Due to the strict focus on ex-post legislative evaluations, the term ‘post-legislative scrutiny’ fell outside the scope of included data. This term was manually searched in all three databases, after which eight studies were added to the dataset (three systematic research studies, three case studies and two expert opinions). The relevant literature (n = 48) was schematically mapped on the basis of which the categories were created.

## Results

The included literature shows that there is a longer history of ex-post legislative evaluations, as over half of the articles were published between 1990 and 2015, slightly less than half were published between 2015 and 2022. The studies show a strong focus on European countries, and the EU, and mainly focus on specific fields of law, such as, health, criminal, EU, and administrative law. The studies written in English mostly relate to post-legislative scrutiny, and EU law. The studies written in Dutch related mostly to health, criminal, and administrative law (one study was related to family law).

Since the ex-post legislative evaluations are conducted in different countries, the context in which an ex-post legislative evaluation takes place differs (van Aeken, 2011).

There are many factors that determine the context, including how countries include the ex-post legislative evaluation into their parliamentary practices, and the different executors of ex-post legislative evaluation studies (such as the ministry, parliament or independent researchers). Depending on the country’s evaluation system, the evaluation is conducted by different parties such as the government, parliamentary committees or external researcher groups. In the Dutch system, for example, ex-post legislative evaluations are conducted by independent researchers outside parliament, while in the United Kingdom, they are also conducted by parliamentary committees (Caygill, 2019a). The influence this difference has on the impact of ex-post legislative evaluations goes beyond the scope of this article. Further in-depth research is therefore necessary in terms of factors that could influence the impact of ex-post legislative evaluations.

Despite these different contexts, similar types of impact have been described in the reviewed literature. These are extracted in this results section and divided into seven categories. A systematic mapping of the literature reveals that older literature on this topic mainly contains opinions on the impact of ex-post legislative evaluations. Several case studies and systematic research studies have taken place in more recent years.

### Knowledge and understanding of the effects of the law

The first type of ex-post legislative evaluation impact identified in the included literature is the knowledge and understanding of the effects of the law in practice (Scheltema, 2002), acquired primarily by the commissioner of the legislative evaluation (such as the ministry, parliament or politicians). Different forms of knowledge are cited in the literature, such as: knowledge about economic and social impact of policies (van Schagen, 2020); fulfilment of policy objectives; the implementation of legislation; the need for legislative amendments; legislative bottlenecks and knowledge of the relationship with other legislation (Hendriks, 2000; van Schagen, 2020; Verschuuren & van Gestel, 2009; Winter, 1997; Winter, 2002; Zwaan et al., 2016). Additionally, systematic research on the implementation and use of legislative and policy evaluations in the Netherlands emphasises that evaluations also bring knowledge to the surface about the implicit assumptions on which the law or policy rests (Klein-Haarhuis & Parapuf, 2016). This subsequently leads to policy transparency in the public domain (Anglmayer & Scherrer, 2020).

At the European level, ex-post legislative evaluations of European legislation lead to more knowledge on the implementation of legislation by Member States (Mastenbroek et al., 2015).

Early publications mention knowledge of evaluation results as the most substantive function of evaluation research (Veerman, 1991). This is in line with early systematic research on the use of ex-post legislative evaluations in the Netherlands, which showed that perusal is the most common form of use of evaluation results (Winter et al., 1990). More recent studies, however, highlight the specific functions of knowledge and insight from legislative evaluations, such as stimulating interaction between the professional players in the field, such as the legislature, the parliament, the judge and the administration (Hendriks, 2000; Scheltema, 2002), reducing uncertainties about the operation of the law in practice (Veerman, 2014), or informing the legislature on the basis of which legislative decisions can be made in similar situations (Nelen, 2000; Winter, 1997). Although several studies mention that ex-post legislative evaluations also provide knowledge about issues in the implementation of legislation (Doust & Hastings, 2019), systematic research on post-legislative scrutiny in the United Kingdom shows that only a small number of recommendations call for action with regard to the implementation of legislation (Caygill, 2019b).

Another function of knowledge acquired from ex-post legislative evaluations described in the included literature is the insight provided as a review mechanism for other forms of advice. For example, ex-post legislative evaluations were used to test the quality of the Dutch Council of State’s advice. This Council provides a form of ex-ante evaluation of upcoming legislation. Information from ex-post evaluation is used to assess the framework and the working method of the Legislative Advisory Council on legislative proposals (Vranken & van Gestel, 2008).

Furthermore, acquiring knowledge and understanding from ex-post legislative evaluations is not only relevant at the individual national or European level, but can also have cross-border relevance. For example, the literature notes that Dutch legislative evaluations on ethical issues, like the Dutch Termination of Life on Request and Assisted Suicide (Review Procedures) Act, also have an effect outside the Netherlands. Other countries, such as Belgium, Italy and France closely follow the practice of euthanasia in the Netherlands, taking into account the knowledge from ex-post legislative evaluations (De Goeij, 2007).

### Confirmation on well-functioning legislation

The second type of impact of ex-post legislative evaluations identified in the included literature is the confirmation of well-functioning legislation. Evaluation results can show that (a part of) a law works properly and thus confirm the intended legislative ideas of policymakers and legislators. Only a few studies included in this scoping review recognise the ability of evaluations to confirm the effectiveness of legislation. For example, one case study concluded that the evaluation had shown that the law had been applied in a careful manner (De Goeij, 2007). In another case study on the ex-post evaluation of the Dutch General Administrative Law Act, the author stated that while confirmation of a well-functioning law (on certain points) is important, it is a less spectacular research result (Michiels, 2010). However, according to the author, such confirmation does not detract from a successful evaluation (Michiels, 2010). A later systematic research on the theory of legislation in the Netherlands (examining 74 legislative evaluations) concluded that legislation in general achieves its goals to a very decent degree (Veerman et al., 2013). Besides achieving the purpose of the law, compliance with the law also scored high in this study. A later study of post-legislative scrutiny in the United Kingdom also mentioned that evaluating legislation can lead to the conclusion that little or nothing needs to be changed (Norton, 2019) – which implies that legislation is functioning well.

### Legislative revision

Legislative revision (also referred to as legislative amendment) is the third type of impact of ex-post legislative evaluations as identified in the included literature. Over the years, several authors have concluded that ex-post legislative evaluations can improve the quality of the evaluated legislation through instrumental use of the evaluation results (European Court of Auditors, 2018; Klein-Haarhuis & Parapuf, 2016; Scheltema, 2002; van Humbeeck, 2000a; van Voorst & Zwaan, 2019; Veerman, 2014; Winter, 1997). Ex-post legislative evaluations contribute to this quality by keeping legislation simple, consistent and up to date (European Commission, 2005; OECD, 2020). Moreover, evaluation results can lead to modification of the law, but also to its abolition (Legemaate, 1997; Veerman, 1991). There are many reasons for this; for example, the results may uncover flaws in a law (Legemaate, 1997) or detect recent societal and/or medical-technological developments that may reveal the need to adapt legislation to new circumstances (De Goeij, 2007). According to one author, it is usually the parliament, ministry or one or more political departments that is particularly interested in evaluation results which can be used concretely in terms of legislative amendments (Veerman, 1991). Other authors also refer to concrete use when legislative amendments are actually implemented (Veerman, 1991; Winter et al., 1990). However, as shown in a case study on Danish and Dutch parental responsibility laws, implemented legislative amendments can be less extensive than recommended in the ex-post legislative evaluation (Jeppesen de Boer, 2014). In fact, researchers can make farfetched recommendations that are little supported by evaluation research (Winter, 1997). In addition, evaluation result recipients can ignore or reject recommendations to amend the law (Nelen, 2000). This is supported by the results of a case study on post-legislative scrutiny in Western Australia, which showed that of all the recommendations from four case studies, only 11% were implemented (Doust & Hastings, 2019). Systematic post-legislative scrutiny in the parliament of the United Kingdom also produced similar results, with only 20% of recommendations in need of amendments being implemented in full or in part (Caygill, 2019a). However, this is not an entirely new insight; previous research has often shown that legislative evaluations do not often lead to legislative amendments, nor to significant changes. For example, systematic research has shown that of more than half of the 35 ex-post legislative evaluations examined resulting in legislative amendments, these concerned relatively marginal amendments of procedural or organisational provisions (Winter et al., 1990). The same conclusion was made on the basis of five case studies that were also conducted in this study. The legislative amendments mainly clarified and refined the legal texts (Winter et al., 1990). Another author, however, criticised this conclusion, alleging that the research results were based primarily on a survey of policymakers, legislative lawyers, and politicians – saying more about their perspectives than about the relationship between evaluation results and the impact on legislative amendments (Nelen, 2000). A later case study on the evaluation of the Dutch General Administrative Law Act also emphasises that the already limited number of recommendations addressed to the legislature has a limited impact on legislative amendments (Michiels, 2010). However, the author noted that it is not easy to determine the extent to which amendments are fully or partly a direct or indirect result of the ex-post legislative evaluation. Moreover, it should be noted that recipients of legislative evaluations are not required to implement legislative evaluation recommendations, but must be open to this. Otherwise the evaluation will miss its target (Legemaate, 1997).

### Influence on the legislative process

In addition to improving legislative texts, several authors mention that ex-post legislative evaluations can also improve the legislative process itself (Gevers, 1995; Hendriks, 2000; Mansaray, 2019; Michiels, 2010; van Humbeeck, 2000a). This is not about the amendments made to the existing legal text but about the process of learning on the role of legislation in society. One author believes that the evaluation process is often more important, as it contributes to the quality of legislation more than the outcome of the evaluation itself. The process leads to gradual understanding of the problems and possible solutions by conducting analyses during the evaluation process (van Humbeeck, 2000a). Other authors see legislative evaluations as part of the legislative process (Eijlander, 1993; Veerman, 1991, 2014). According to these authors, this process should not only focus on the preparation of new legislation but also on the evaluation of existing legislation and regulations in terms of effectiveness and relevance. By being open to lessons learned, contributions can influence the continuation of a good legislative process (Mansaray, 2019). They can also create the possibility to retrospectively test the assumptions of a law (Winter, 2002). Moreover, ex-post legislative evaluations can contribute to the appropriate use of the legislation instrument (Gevers, 1995) because it enables politicians during the legislative process to make considerations based on reliable information about the operation of the law in practice. This enhances the quality of a good debate during the legislative process (Winter, 1997) and may also help to prevent previous mistakes made by the legislature (Hendriks, 2000). In this way, legislative evaluations can have a potential deterrent effect that make policy makers think twice before introducing new legislative proposals (Norton, 2019).

In some countries (and especially at the European level), ex-ante legislative evaluations are also part of the legislative process. Findings of ex-post legislative evaluations are often used in the regulatory impact assessments conducted to inform amending proposals (Poptcheva, 2013). Insights derived from ex-post legislative evaluations into the effects of a law or regulation serves as important input for ex-ante legislative evaluations and the drafting of new laws and regulations (Verschuuren & van Gestel, 2009; Zwaan et al., 2016). This has been demonstrated by systematic research on the use of evaluative information in the European Union (van Golen & van Voorst, 2016). Half of the 225 studied ex-ante legislative evaluations on proposals for legislative amendments used available information from ex-post legislative evaluations (van Golen & van Voorst, 2016). Using ex-post legislative evaluations results in ex-ante legislative evaluations and vice versa can assess and improve the quality of ex-ante legislative evaluations and, thus, lead to a learning loop in the European regulatory cycle (Verschuuren & van Gestel, 2009).

In this regard, ex-post legislative evaluations can be used at the final stage of the regulatory cycle (European Court of Auditors, 2018; Klein-Haarhuis & Parapuf, 2016) to assess the implementation of European legislation by Member States (Mastenbroek et al., 2015) or to improve the effectiveness of European legislation with legislative amendments (van Voorst, 2018). Yet, the European Court of Auditors describes ex-post legislative evaluations as “a key element of the EU policy cycle as it contributes to the better regulation cycle” (p. 33) (European Court of Auditors, 2018). The important role that ex-post legislative evaluations play in the European legislative process is also evident from it forming part of the ‘Better Regulation Agenda’, launched in 2015 by the European Commission (European Court of Auditors, 2018). Before revising or introducing new legislation, the European Commission prioritises the evaluation of existing legislation, also referred to as the ‘evaluate first principle’, in place since 2013 (European Court of Auditors, 2018). This is supported by systematic research of the European Court of Auditors, where 27 of the 32 legislative initiatives were based on ex-post evaluations (European Court of Auditors, 2018). The European Commission intended to adhere strictly to the ‘evaluate first’ principle, since “Already, over 80% of the Commission’s impact assessments supporting legislative revisions are based on an evaluation.” (p. 17) (European Commission, 2021). Although the Commission seems to make proper use of ex-post legislative evaluation results, the European Commission has stated that the European Parliament, the Council and the national actors make too little use of the insights resulting from ex-post legislative evaluations (van Schagen, 2020).

### Influence on the policy process

The fifth type of impact of ex-post legislative evaluations identified in the included literature is the influence ex-post legislative evaluations have on the policy process. Although the policy process and the legislative process share many characteristics and sometimes overlap, the former is much broader in scope as compared to the legislative process. According to various authors, the policy process can be influenced by ex-post legislative evaluations (Vanlandingham, 2011; Winter, 1997) due to the knock-on effects of evaluation results in improved policies or policy decision making (Eijlander, 1993; van Aeken, 2018; van Humbeeck, 2000a). The evaluation results provide a better understanding on the effects of different alternatives (van Humbeeck, 2000b) and enable choices to be made for or against certain policy options in a more objective and transparent manner (van Schagen, 2020). Both new and existing policy decisions can be adjusted on the basis of ex-post legislative evaluation results (Nelen, 2000) as they can, for example, lead to agenda-setting on the basis of which the process of amending the law can be initiated (Anglmayer & Scherrer, 2020). However, the evaluation of legislation only serves to support policy-making and cannot replace political decision-making. In addition, some authors noted that legislative evaluations sometimes have a minor impact on the policy process (Veerman, 1991) or final policy decisions (van Humbeeck, 2000b).

From another perspective, final legislative evaluations as well as ongoing ones can contribute to the policy process, as mentioned in a publication on the utilisation of evaluation results in legal policy-making and administration (Wollmann, 2017). The ongoing evaluation process may create the opportunity to rectify and modify a policy design or implementation process based on interim evaluation results (Wollmann, 2017).

Several authors also mention the oversight and accountability function of ex-post legislative evaluations in the policy process at both the national (Doust & Hastings, 2019; Mansaray, 2019; Winter, 1997) and European levels (Anglmayer & Scherrer, 2020; European Commission, 2021; van Voorst, 2018). ex-post legislative evaluations can act as an executive oversight tool (Griglio, 2019) by increasing government accountability, thereby adding value to the Parliament’s oversight role (Mansaray, 2019). One case study also suggests that “Overall, the fact that the European Parliament’s ex-post evaluations have, in a number of cases, been successful in influencing or informing the Commission’s policy cycle could encourage other national parliaments to expand their own evaluation activities, from a passive to a more active role, in order to possibly strengthen their oversight function.” (p. 423) (Anglmayer & Scherrer, 2020)

Systematic research of Zwaan et al. (2016) on the usage of ex-post legislative evaluations of European Union legislation by members of the European Parliament for holding the Commission political accountable presents an analysis of 220 evaluations. The authors examined the number of evaluations that were followed up via parliamentary questions posed to the Commission, concluding that 49 evaluations were followed up on -and mostly with forward looking agenda-setting and policy change purposes, rather than accountability purposes (Zwaan et al., 2016). In total, 34 evaluations were used to steer the behaviour of the Commission. However, the members of the European Parliament seemed particularly interested in the actions to be taken rather than in exposing the European Commission’s shortcomings (Zwaan et al., 2016).

### Influence in the political sphere

The sixth type of impact of ex-post legislative evaluations identified in the included literature is the impact of ex-post legislative evaluations in the political sphere. Several authors stated that ex-post legislative evaluations can start a political discussion on certain topics, such as the objectives of a law and the balancing of interests enshrined in it (De Goeij, 2007; Gevers, 1995). Ex-post legislative evaluations of ethically sensitive legislation (such as the Dutch laws on abortion and euthanasia) frequently lead to political and social discussions which ensures that evaluation results are taken seriously (De Goeij, 2007). In addition, evaluation results can help to increase the quality of democratic deliberation with plausible arguments (Bussmann, 2010); resolve political conflicts (Eberli, 2018); or clarify and give more weight to political discussions or decisions at both national and European levels by providing support with evaluation data (Eberli, 2018; van Voorst, 2018; Veerman, 1991; Winter, 1996). Evaluation data can also influence existing political positions, as demonstrated by early research on five case studies on Dutch regulations (Winter et al., 1990). These case studies examined whether the findings of the evaluation report were considered by political actors. The results showed that there was evidence of positional influence on political actors in all five case studies, and only to a lesser extent in one of these five case studies (Winter et al., 1990).

Evaluation results can also tactically be used in the political sphere. Politicians can use evaluation data to defend decisions that have already been made (van Humbeeck, 2000a); delay a decision (Eberli, 2018); or hide behind the data when legislative results are disappointing (Nelen, 2000). In this way, legislative evaluations have a legitimising or justificatory function (Eijlander, 1993). Moreover, the political debate following an ex-post legislative evaluation can also lead to selective use of evaluation results by politicians (Gevers, 1995; Veerman et al., 2013). After all, there is always a risk that legislative evaluations will produce results other than those expected, giving new ammunition to the opponents of those who requested the evaluation (Nelen, 2000). Actors who feel threatened by those evaluations may, for political reasons, try to prevent the use of evaluation results or selectively use results that fit their agenda (Veerman, 1991; Veerman et al., 2013). This was also shown in a publication on the fitness check of European consumer law in which the author compared an evaluation study conducted by a consulting company with the subsequent commission report (van Schagen, 2020). The author conclude that the Commission selectively used the evaluation results by omitting a critical point about unclear general conditions from the evaluation study in the Commission report (van Schagen, 2020).

Another tactical form of using ex-post legislative evaluations is to let them function as political bargaining to win over parliamentarians who oppose a law that has yet to pass (Veerman, 2014). However, as demonstrated in a case study on the use of ex-post legislative evaluations by the European Commission, such opposition by key political actors at the European level does not stop the instrumental use of ex-post legislative evaluations (van Voorst & Zwaan, 2019). At the national level, the insertion of an evaluation clause in the law sets as a condition that the law will be discussed again after entering into force (Veerman, 1991; Vranken & van Gestel, 2008; Winter, 1996). A potential risk here is that the political landscape may change by the time the evaluation is due (according to the evaluation clause) (Legemaate, 1997).

An example is described in a case study on the Dutch Directors’ Liability Act, where the House of Representatives doubted the usefulness of this act and its burden on business. The Minister of Justice proposed conducting an evaluation in order to ensure a majority for the bill (Veerman, 1991).

An additional political strategic advantage of ex-post legislative evaluations may be to win time, to get the issue off the table for a while so that it is no longer on the political agenda (Veerman, 1991). This can also ensure that the interlocutors (and their constituencies) can get used to each other’s political positions (Veerman, 1991).

### Influence on society

In addition to having an impact on the formal participants in the ex-post legislative evaluation process, evaluation results may also have impact on society. Laws have a codifying and modifying character. As such, they have become more instrumental in modifying social behaviour, offering certain guarantees, to society, and in influencing it overall. Ex-post legislative evaluations can provide insight on the extent to which legislation matches citizens’ perceptions (De Goeij, 2007), and could as a result “(..) provide an important link between citizens and parliament, but may not always live up to its promise.” (p. 2) (Moulds & Khoo, 2020). However, despite it being clear that legislation directly affects society, only a few authors indirectly mention the impact of ex-post legislative evaluations on society. In earlier research, the literature pointed to there being an opportunity for citizens to benefit from evaluation results as potential users of legislative evaluations (Hendriks, 2000; Poptcheva, 2013). The authors claimed that evaluation results could, for example, influence societal opinions, lead to debates among the citizenry, and strengthening the democratic debate. Since evaluation results are accessible to any interested party, including citizens and the media, they ensure transparency in the public domain, which means that people are better informed (Anglmayer & Scherrer, 2020; Norton, 2019).

Van Aeken (2018) has conducted a surprising addition to the body of research in the form of a conceptual exercise, examining whether ex-post legislative evaluations could also contribute to the democratic process. The author noted that a shift from government to governance in Belgium presented an opportunity for the democratic functionality of an ex-post legislative evaluation (among other things), but concluded that there are no signs of political support for this (van Aeken, 2018). Besides direct effects, ex-post legislative evaluations also have indirect effects on society. One study affirmed, for example, the importance of involving citizens in the implementation of ex-post legislative evaluations in an effort to rebuild trust between citizens and institutions (Moulds & Khoo, 2020). A different perspective was given in the OECD’s policy outlook describing the importance of ex-post legislative evaluations in relation to the COVID-19 pandemic. It emphasised that knowledge from ex-post legislative evaluations about what has and has not worked or could be improved is crucial for improving future well-being and, thus, has an indirect impact on society (OECD, 2021).

## Discussion

This scoping review shows that different types of actual and potential impact of ex-post legislative evaluations can be retrieved from the literature. Although the literature makes no explicit distinction between types of impact of ex-post legislative evaluations, reviewing the included studies in this study resulted in the following categories: 1) knowledge and understanding of the effects of the law, 2) confirmation of well-functioning legislation, 3) legislative revision, 4) influence on the legislative process 5) influence on the policy process, 6) influence in the political sphere, and 7) influence on society. The different types of impact of ex-post legislative evaluations relate in varying degrees to the different parties involved and different stages in the legislative process. Strikingly, a comprehensive understanding of the first category must be completed before moving onto any of the subsequent categories. Knowledge and understanding can be seen as a prerequisite for the other types of impact, as they relate to a form of exploitation of the knowledge and understanding derived from ex-post legislative evaluations. In addition, the other types of impact (numbers 2–7) are interrelated and are more or less existing perspectives that are expressed in the material reviewed for this study. However, they are not mutually exclusive because of their interconnectedness.

Despite the different contexts in which ex-post legislative evaluations take place due to the different evaluation systems that exist in every country, similar types of impact can be found in every country. Thus, the seven categories are relevant to a wide range of ex-post legislative evaluations. With this study, we aim to contribute to existing literature on ex-post legislative evaluation usage, but also to develop a better understanding of the different types of impact. In addition, the research results show that there is room for more in-depth, follow-up research into the factors that influence the impact of ex-post legislative evaluations. This scoping review shows, for example, that there are different parties who conduct ex-post legislative evaluations, and different conditions amidst which evaluations take place. It would be interesting to examine to what extent these differences lead to different types or degrees of impact of ex-post legislative evaluations.

Earlier ex-post legislative evaluation research has already shown three phases of ex-post legislative evaluation use, including: gaining knowledge, influencing political points of view and legislative knock on effects (Winter et al., 1990). According to one of the authors in a later dissertation study, these concern three successive phases (Winter, 1996). The first phase -knowledge-, is a necessary prerequisite for the following two phases because without knowledge, there can be no influence on political points of view, or knock-on effects of evaluation results in legislative amendments (Winter, 1996). This is in line with the insight derived from this study: that knowledge and understanding is a requisite to achieve one of the other categorised types of impact. Furthermore, the author refers to seven models of use distinguished by another author (Weiss, 1979), from which the following three main categories are derived: specific use, generic use and tactical use. Winter et al. (1990) conducted five case studies and a meta-analysis in which 35 legislative evaluations are studied from these three phases of use. Since this study was conducted in 1990, the current scoping review also includes more recent literature, and thus adds more recent insights to this earlier study. This scoping review found similar types of impact (e.g. knowledge and understanding, policy influence and improved legislative texts); tactical use also emerged in this study. However, this scoping review distinguishes additional forms of impact being: confirmation of well-functioning legislation, influence on the legislative and policy process, political debate and society.

A later dissertation study on post-legislative scrutiny in the UK parliament mentioned three different areas of impact (of these evaluations): stopping or persuading the government to not take action, putting the issue on the political agenda and a lack of impact all round (Caygill, 2019a). These areas are recognisable, but not exhaustive. They are also based on the degree to which recommendations from post-legislative scrutiny were accepted, which can be considered a limited scope. In contrast, our study used a broad scope, looking at all ex-post legislative evaluations in different countries. From this we have been able to obtain a more complete picture of the various types of impact of ex-post legislative evaluations.

To begin with, only a few studies point out that ex-post legislative evaluations address the succeeding of a law. Yet, surprisingly, such result is described as ‘less spectacular’. This could be seen as a remarkable description, since knowing what works well should, in our view, also be considered a spectacular result, as it is a positive affirmation of the proposed policy and legislative decisions. Nevertheless, the reviewed literature shows a clear focus on making amendments to legislative texts based on ex-post legislative evaluations. Moreover, some literature seems to indicate that evaluations are successful only if the results show that amendments to the law are needed. These amendments would subsequently improve the quality of legislation. The legislators or policy makers would be particularly interested in evaluations with a view to concrete legislative amendments. In most cases, however, these are minor amendments. Therefore, we believe that, for ex-post legislative evaluations to contribute to a learning cycle, it is essential not to focus on required amendments only, but also on what was successful. Whether or not a law needs to be amended may be revealed by the evaluation results, but should not be considered the only starting point. Being open to all possible effects can provide a better picture of the efficacy of the law in practice.

The strong focus on the impact of ex-post legislative evaluations on the law itself and the legislative process confirms the specificity of legislative evaluations as compared to policy evaluations. Given this observation, whether this impression is a fair representation of reality or a biased representation by those writing on this subject could be questioned: is the focus on the law itself a characteristic of legislative evaluations or of those writing about these evaluations? In one of the included publications, the author stated that “The legal system is in the end not concerned with budgets and effects, but with legitimacy, and ultimately with fairness and justice.” (p. 286) (van Aeken, 2018). This confirms the focus on legal aspects in ex-post legislative evaluations instead of broader effects. On the other hand, another author (Nelen, 2000) also notes that a narrow view on impact may lead to a bias in reporting on the impact of legislative evaluations, for example, by involving only officials working within the legislative process in a survey study, which could lead to their perceptions being labelled as the empirical truth. These observations raise the question about whether this provides a realistic overview of the impact of ex-post legislative evaluations, or a more selective overview. It is important that future evaluations and reflection on this subject move away from a focus on merely the impact of ex-post legislative evaluations on minor legislative amendments, and instead move towards having a broader perspective.

The impact of ex-post legislative evaluations on society is an equally important factor, and yet the effect of legislative evaluations on society does not seem to be a key point of attention. However, ex-post legislative evaluations can ultimately have an impact on society, as citizens are the implementers of the law in practice (for example by giving substance to self-regulation). Legislative evaluations can have an instrumental impact on the daily life of citizens by influencing rules and arrangements and by strengthening or undermining the legal position of citizens. Since citizens are also important stakeholders in this domain, it would be fundamental for future research to include the influence and role of legislative evaluations in society. How they may benefit or what impact a legislative evaluation may have is, however, not examined or described in the reviewed literature.

This scoping review also shows that at both national and European levels, ex-post legislative evaluations can be used tactically in the political and legislative debate. Ex-post legislative evaluations could, for example, function as political bargaining tools to get a bill passed, postpone the political discussion, win time or to defend decisions already made. In this way, selective use can be made of the evaluation results, (which is often referred to in the literature). Actors who feel threatened by legislative evaluations may try to prevent the use of such results or perhaps opt only to selectively use those results that fit their own agenda. It is worth questioning whether tactical intentions do not overstep the bounds of the objectives of ex-post legislative evaluations, as they should be instruments to open eyes, not to hide behind or use selectively.

A noteworthy limitation of this study is that not every form of impact is visible because the impact can take longer to be appear or is either not equally visible or not measurable (Norton, 2019). It can sometimes be unclear whether something is a direct result of a legislative evaluation. Another limitation of this study is that some of the included literature does not focus solely on ex-post legislative evaluations, but has a broader scope and thus also includes policy evaluations. The second important limitation of this study is that most authors speak of potential effects of ex-post legislative evaluations. These are not based on conducted empirical research. Despite a proportionate distribution of the different types of included publications, mainly expert opinions deal with the different forms of impact. This is supported to a lesser extent by case studies or systematic research. As a result, it is hard to determine whether the expert opinions can be substantiated beyond the author’s experience. On the other hand, including both studies on potential and on actual types of impact make it possible to compare these two types of studies in this scoping review.

## Conclusion

The research findings in this scoping review presented an overview of the literature’s focus on the impact of ex-post legislative evaluations. The literature shows that the impact of ex-post legislative evaluations cannot be described as a unambiguous concept. There is no such a thing as ‘the’ impact of ex-post legislative evaluations, but there are several types than can range from informational to law-changing and can take place in different domains such as in politics or in society.

However, although legislation always has an impact on society, this scoping review shows that little is written about whether ex-post legislative evaluations also have an impact on society. The literature focuses on legislative revision and tactical use within the political sphere.

As the knowledge base for this scoping review mainly consists of expert opinions and case studies, more extensive empirical research could contribute to a more validated insight in the impact of ex-post legislative evaluations.

This scoping review is limited to the question of what types of impact of ex-post legislative evaluations are described in the current literature. The resulting question is what factors influence the realisation of these different types of impact. A closer analysis of the actual impact of ex-post legislative evaluations or the factors that influence the impact of ex-post legislative evaluations would therefore be an interesting step for further research. The insights gained from this scoping review provide valuable starting points for future research because it invites one to move away from the current narrow view on legislative amendments and open a broader view in future studies.

## Figures and Tables

**Table 1:** Final scientific search strategies.

**Web of Science, Worldcat Discovery and Legal Intelligence**
***Dutch search terms***
*1st strategy*
TI = (wetsevaluatie* OR ‘Evaluatie wet*’ OR ‘evaluatie regel*’)
*2^nd^ strategy*
TI = (wetsevaluatie* OR ‘Evaluatie wet*’ OR ‘evaluatie regel*’ AND aanpak OR uitvoering OR method*)
*3^rd^ strategy*
TI = ((wetsevaluatie* OR evaluatie wet* OR evaluatie regel*) AND (impact OR gevolg OR invloed OR effect**))
***English search terms***
*1st strategy*
TI = (‘Legislative evaluation*’ OR ‘Law evaluation*’ OR ‘Evaluation of legislation*’ OR ‘Legal evaluation*’)
*2nd strategy*
TI = ((legislative evaluation* OR law evaluation* OR evaluation of legislation OR legal evaluation*) AND method*))
*3rd strategy*
TI = ((legislative evaluation* OR law evaluation* OR evaluation of legislation OR legal evaluation*) AND (impact OR influence OR result* OR utilization))

## Note

1 Post-legislative scrutiny is defined by the Law Commission of England and Wales as “A broad form of review, the purpose of which is to address the effects of legislation in terms of whether intended policy objectives have been met by the legislation and, if so, how effectively. However this does not prevent the consideration of narrow questions of a purely legal or technical nature.” (p. 7) (The Law Commission, 2006).
